# An Integrated Approach toward the Biomanufacturing of Engineered Cell Therapy Products in a Stirred-Suspension Bioreactor

**DOI:** 10.1016/j.omtm.2018.04.007

**Published:** 2018-04-27

**Authors:** Charlie Y.M. Hsu, Tylor Walsh, Breanna S. Borys, Michael S. Kallos, Derrick E. Rancourt

**Affiliations:** 1 Department of Biochemistry and Molecular Biology, Cumming School of Medicine, University of Calgary, Calgary, AB T2N 4N1, Canada; 2 Pharmaceutical Production Research Facility, Schulich School of Engineering, University of Calgary, 2500 University Dr. NW, Calgary, AB T2N 1N4, Canada; 3 Biomedical Engineering Graduate Program, University of Calgary, 2500 University Dr. NW, Calgary, AB T2N 1N4, Canada; 4 Department of Chemical and Petroleum Engineering, Schulich School of Engineering, University of Calgary, 2500 University Dr. NW, Calgary, AB T2N 1N4, Canada; 5 Department of Oncology, Faculty of Medicine and Dentistry, Cumming School of Medicine, University of Calgary, Calgary, AB T2N 4N1, Canada; 6 Department of Medical Genetics, Cumming School of Medicine, University of Calgary, Calgary, AB T2N 4N1, Canada

**Keywords:** bioprocess, gene delivery, transfection, biotechnology, cationic polymer, cell therapy, biomanufacturing, genetic engineering, plasmid DNA, bioreactor

## Abstract

Recent advances in stem cell biology have accelerated the pre-clinical development of cell-based therapies for degenerative and chronic diseases. The success of this growing area hinges upon the concomitant development of scalable manufacturing platforms that can produce clinically relevant quantities of cells for thousands of patients. Current biomanufacturing practices for cell therapy products are built on a model previously optimized for biologics, wherein stable cell lines are established first, followed by large-scale production in the bioreactor. This “two-step” approach can be costly, labor-intensive, and time-consuming, particularly for cell therapy products that must be individually sourced from patients or compatible donors. In this report, we describe a “one-step” integrated approach toward the biomanufacturing of engineered cell therapy products by direct transfection of primary human fibroblast in a continuous stirred-suspension bioreactor. We optimized the transfection efficiency by testing rate-limiting factors, including cell seeding density, agitation rate, oxygen saturation, microcarrier type, and serum concentration. By combining the genetic modification step with the large-scale expansion step, this not only removes the need for manual handing of cells in planar culture dishes, but also enables the biomanufacturing process to be streamlined and automated in one fully enclosed bioreactor.

## Graphical Abstract

**Figure:**
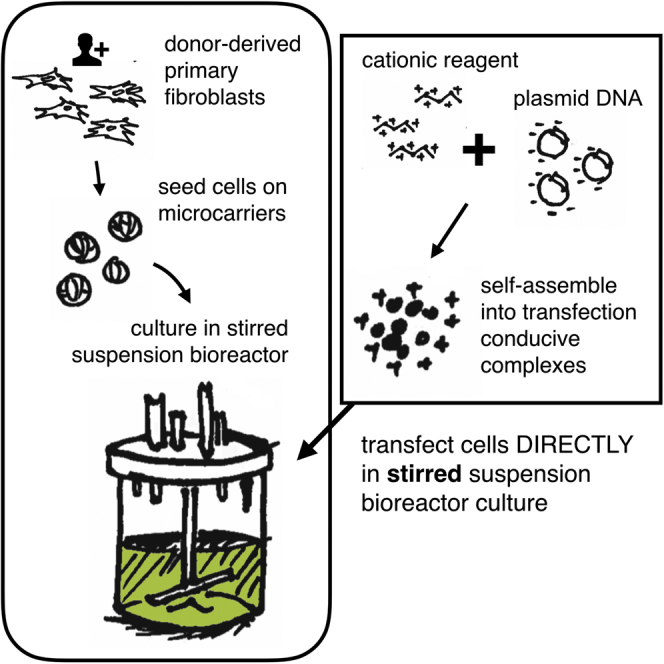

## Introduction

Recent advances in our understanding of stem cell biology and the cues to direct them into their functional derivatives have accelerated the pre-clinical development of stem cell-based therapies for the treatment of a wide range of chronic and degenerative diseases.^1^, ^2^, ^3^ However, one of the biggest technical hurdles that must be overcome for stem cell-based therapies to become a clinical reality is the large-scale manufacturing of high-quality, well-characterized, affordable cell-based products.

Current cell manufacturing platforms are built on the production model established for vaccines and biologics, which has several fundamental differences compared to cell therapy products (CTPs). For one, cells grown for biologics are only vehicles for production, whereas for cell therapies, the end product is the cell itself, which can respond to the physicochemical factors in the bioreactor and become significantly altered during the scale-up process. Second, CTPs cannot be produced from a single cell source and need to be matched to the patient. This requires production from multiple sources of starting material. As such, there is often inherent heterogeneity in terms of efficacy, quality, and yield of the product.^4^ Finally, production of biologics often requires extensive cell line development to generate stable high-expression clones. This is a lengthy and laborious process that might not be needed for reprogrammed cell therapy products that typically only require transient forced expression of reprogramming factors to re-direct cell fate,^5^, ^6^ induce pluripotency,^7^, ^8^ or convert cell type.^9^, ^10^ These fundamental differences in the way that biologics and cell therapy products are sourced and engineered point toward a need for a new biomanufacturing paradigm that can be scaled up to meet quantity demand and scaled out to individualize the treatments for different cohorts of patients.

One solution to increasing the scale and speed of production for genetically engineered CTP is to integrate the derivation and expansion steps into one continuous bioprocess by direct transfection of cells in a bioreactor. Transduction by viral vectors such as gamma-retrovirus, lentivirus, and adenovirus are among the most widely adopted gene transfer approaches due to their efficiency and versatility.^11^, ^12^ However, immunogenicity to viral components and mutagenesis due to ectopic insertion of the transgene can present major safety problems in the highly proliferative stirred-suspension environment.^13^ With recent advances in non-viral gene delivery systems showing efficiency comparable to viral vectors,^14^, ^15^ the cost and safety profile of chemical-based non-viral gene delivery methods makes it well suited for adaptation into scalable manufacturing platforms.

Here, we describe a proof-of-concept toward the development of an integrated bioprocess for the biomanufacturing of genetically engineered cells by combining the genetic modification step with the large-scale expansion step into one continuous stirred-suspension bioreactor platform. A central feature of our approach is the direct non-viral transfection of primary fibroblasts on microcarriers in the bioreactor. This allows the production platform to be scaled and further modularized with different nucleic acid molecules. The bioprocessing platform additionally allows dynamic control of key culturing variables (e.g., pH, dissolved oxygen, stir speed), which can be exploited to modulate cell proliferation rates and metabolic activity, which we show here not only increase the duration of transgene expression, but further increase the level of expression.

## Results

### Evaluating Cationic Reagent Suitable for Transfection in Suspension Culture

Ideally, transfection in the stirred-suspension culture would be performed in growth media to minimize changing and replacing media following transfection. As such, one of the first criteria that we factored into the evaluation of transfection reagent is the ability for cells to be transfected in basal growth media. We focused our evaluation on commercially available reagents in order to make our method more accessible to a broader research community. The list of reagents evaluated in this study are XtremeGENE 9, XtremeGENE HP, JetPrime, TransIT-LT1, TransIT-2020, TransIT-X2, and TransIT-3D, as they exhibit broad spectrum activity and are widely accessible through several distributors. We further included Lipofectamine 2000 and Lipofectamine 3000 for comparison, as these were among the most popular and widely cited lipid-based commercial reagents.

**Figure 1:**
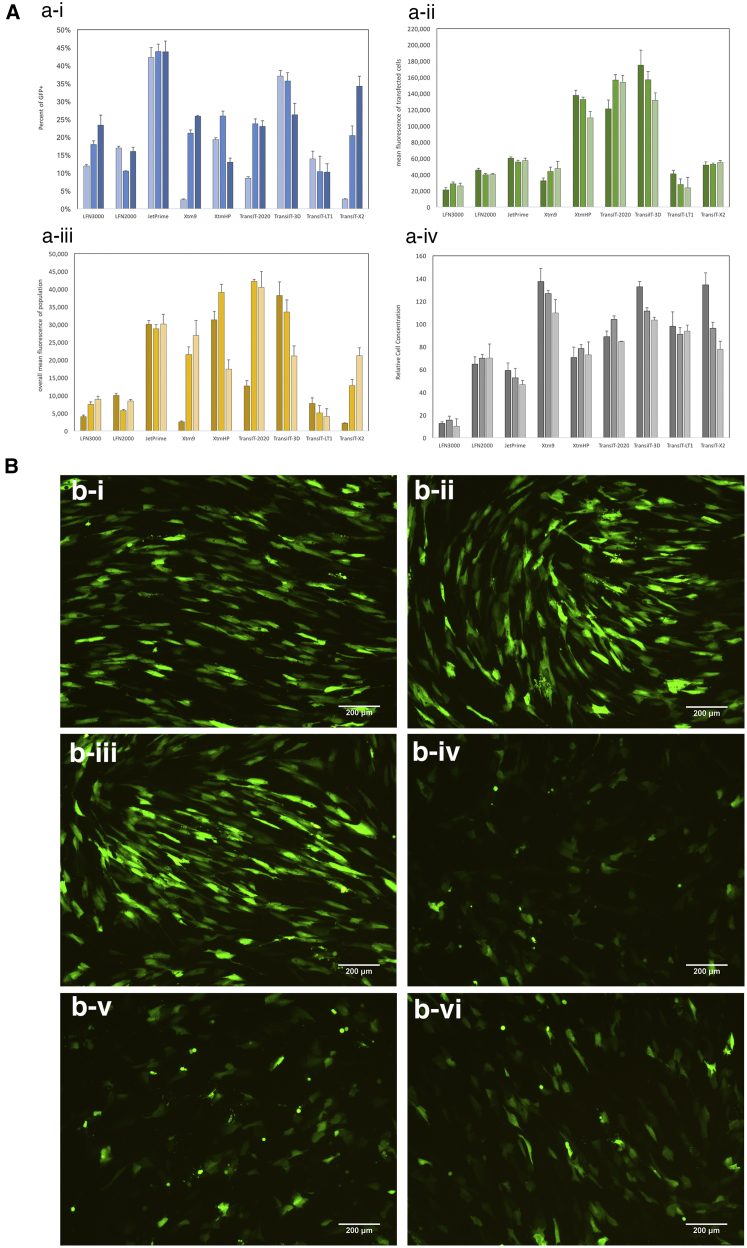
Efficiencies of Commercial Transfection Reagents in Normal Human Foreskin Fibroblast on Planar Culture Dishes (A) Transfection efficiencies of commercial reagents in normal human foreskin fibroblast (NHFF) at reagent-to-pDNA weight ratios of 2 (low), 3 (med), and 4 (high). Transfection efficiencies are collectively represented by (i) the percentage of transfected cells, (ii) the mean fluorescence of transfected cells, (iii) the overall fluorescence of the population, and then factor in (iv) the relative cell number, which is reflective of the reagent’s toxicity. Analyses were carried out by adjusting the gate such that the fluorescent population of NHFF transfected with a control plasmid (i.e., empty vector; gWIZ) is 1%. Data was then normalized against a non-transfected control to adjust for day-to-day variability in autofluorescence. An efficient transfection reagent in this case was defined as high overall mean fluorescence, which are XtremeGENE HP, TransIT-2020, and TransIT-3D (n = 4). Data represent mean ± SD. (B) Representative epi-fluorescence images of HFF transfected with (i) TransIT-3D, (ii) TransIT-2020, (iii) XtremeGENE HP, (iv) JetPrime, (v) Lipofectamine 3000, and (vi) XtremeGENE 9. These images reveal that fluorescence intensity of the transfected cells varies among transfected reagent, even if the percentage of transfected cells are similar, as in the case of JetPrime versus TransIT-3D. As well, significant toxicity can be seen for cells transfected with JetPrime, Lipofectamine, and XtremeGENE 9, as evident by the punctate cell morphology, in contrast to the healthy fiber-like shape. Scale bar, 200 μm.

To accurately assess the relative efficiency of each of the transfection reagents, we transfected cells at various reagent-to-plasmid DNA (pDNA) weight ratios, (v/w; 2, 3, and 4) since optimal ratios at which the highest efficiencies can be achieved for each carrier can differ. Figure 1A shows the relative transfection efficiencies of the transfection reagents. Among the transfection reagents evaluated, XtremeGENE HP, TransIT-2020, and TransIT-3D JetPrime had the highest overall mean fluorescence (Figure 1A-iii), which is a function of the percent of transfected cells (Figure 1A-i), mean fluorescence of the transfected cells (Figure 1A-ii), and relative cell concentration (Figure 1A-iv). In contrast to the non-liposomal cationic reagents, the cationic lipids, Lipofectamine 2000 and Lipofectamine 3000, performed poorly in basal media, yielding less than 25% of the fluorescence intensity as that of the XtremeGENE HP. The differences in transfection efficiencies among the reagents tested were further evident under epi-fluorescent microscopy. Figure 1B shows epi-fluorescence images of human foreskin fibroblast (HFF) transfected with TransIT-3D (Figure 1B-i), TransIT-2020 (Figure 1B-ii), and XtremeGENE HP (Figure 1B-iii), which exhibit long, elongated morphology typical of viable fibroblasts. In contrast, cells transfected with JetPrime (Figure 1B-iv), Lipofectamine 3000 (Figure 1B-v), and XtremeGENE 9 (Figure 1B-vi) exhibited more rounded, punctuated shapes that are more often associated with cytotoxicity. The fluorescence intensity of cells transfected with JetPrime, Lipofectamine 3000 and XtremeGENE 9 are also much dimmer compared to TransIT-2020, TransIT-3D, and XtremeGENE HP, which is consistent with the mean fluorescence values summarized in Figure 1A. Based on the results shown in Figure 1, we chose XtremeGENE HP for our subsequent experiments owing to its consistency in performance among experimental replicates, product availability, cost incentive, and bulk discount.

### Evaluating Microcarriers Suitability for Transfection

Anchorage-dependent cells such as fibroblasts require a solid substrate for growth, so we have employed microcarriers as a scalable culturing substrate. Microcarriers have been widely adopted for the expansion of a variety of adherent cell types in stirred-suspension bioreactors.^16^, ^17^, ^18^, ^19^, ^20^ However, microcarriers come in different surface coatings, charges, densities, sizes, and surface areas,^21^ which altogether can affect cell attachment efficiency, proliferation rate, and compatibility for transfection with cationic complexes. Thus, microcarriers most conducive for transfection need to be empirically evaluated. Table 1 lists the microcarriers screened in this study and their physical properties.

**Table 1:** List of Microcarriers Used in This Study, Their Features, Physical Properties, and Specifications

Microcarrier	Material	Surface Coating	Charge	Density	Size (μm)	Surface Area (cm^2^/g)
Collagen	crosslinked polystyrene, modified with gelatin	type I porcine collagen	no	1.02	125–212	360
FACTIII	crosslinked polystyrene	type I porcine collagen	cationic	1.02	125–212	360
Glass	crosslinked polystyrene, modified with high-silica glass	silica glass	no	1.02	125–212	360
Plastic	crosslinked polystyrene	none		1.02	125–212	360
Plastic^+^	crosslinked polystyrene, cationic	none	cationic	1.02	125–212	360
Pronectin F	crosslinked polystyrene, modified with recombinant fibronectin	recombinant fibronectin	no	1.02	125–212	360
Hillex II	modified polystyrene, modified with cationic trimethyl-ammonium	trimethyl- ammonium	cationic	1.11	160–180	515
Cytodex 3	crosslinked dextran, denatured collagen on the surface	porcine gelatin	no	1.04	141–211	2,700
CultiSphere S	crosslinked pharmaceutical-grade gelatin	porcine gelatin	no	1.04	130–180	7,500

#### Defining the Optimal Time Frame for Transfection Based on Proliferation Rate

**Figure 2:**
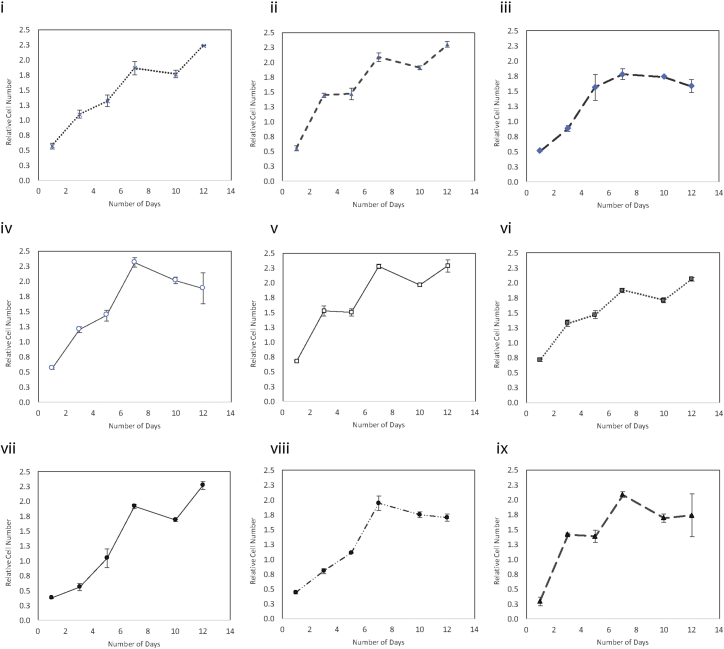
Cell Proliferation on Microcarrier in Static Suspension over a 12-Day Period (i) FACTIII, (ii) collagen, (iii) plastic^+^, (iv) plastic, (v) glass, (vi) CultiSphere S, (vii) Pronectin F, (viii) Cytodex 3, and (ix) Hillex II. Units are expressed as a factor of a reference control, which were a sample of mitotically arrested cells that have been seeded on static culture at the time of experimental setup in order to normalize the measurement for day-to-day variability. In general, highest proliferation rates were observed between days 1 and 5; Hillex II had the large increase in cell number over this period, even though starting cell numbers were the lowest, suggesting that material of the microcarrier may have an effect on cell proliferation rates. Data represent mean ± SD.

Typically for static planar culture, a culture confluence of 80%–90% is optimal for transfection. This is because adherent cultures require cell-to-cell contact for proliferation. Since polymer-assisted transfection relies on the transient breakdown of the nuclear membrane during mitosis for passive nuclear translocation, a highly proliferative culture generally correlates with higher transfection efficiency.^22^, ^23^ However, confluence for suspension culture is difficult to gauge under the microscope, since it is hard to see the microcarrier in its entirety. As well, bead-to-bead contact can facilitate cell migration (bridging), allowing cells to continue to expand even after individual beads appear to be fully confluent. Thus, instead of relying on culture confluence for transfection, we sought to map the growth kinetics of the microcarrier cultures over a course of 12 days to determine the fastest proliferative part of the culture period. Figure 2 shows the growth curves of the nine microcarriers evaluated in this study. Overall, glass (Figure 2-v), CultiSphere (Figure 2-vi), Cytodex D3 (Figure 2-viii), and Hillex II (Figure 2-ix) had the shortest doubling time (fast growth) between days 1 and 3 after initial seeding, which we hypothesize would be the most conducive to transfection. Interestingly, microcarriers with comparable attachment efficiency and hence initial starting cell density do not necessarily result in the same growth rate, as can be seen with FACTIII (Figure 2-i) versus collagen (Figure 2-ii), where the latter had a much sharper slope from days 1 to 3. This suggests that, while cell-cell contact promotes faster proliferation, the surface charge and coating material may also have a tertiary effect on the growth kinetics.

**Figure 3:**
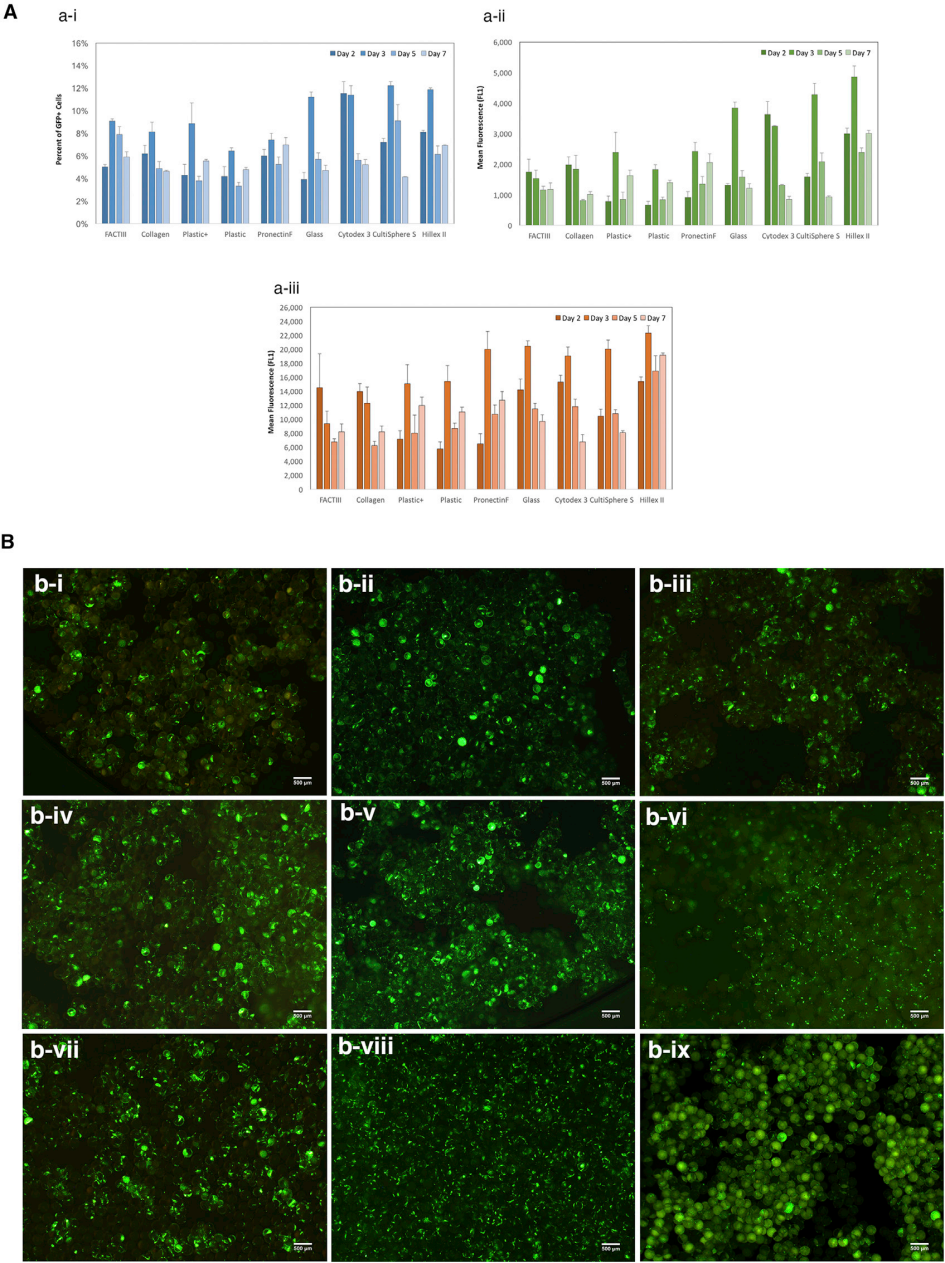
Transfection of NHFF on Microcarriers (A) Transfection optimization as a function of growth curve. Cells were seeded on microcarrier, then transfected on days 2, 3, 5, and 7. Transfection efficiencies are represented by (i) percentage of transfected cells, (ii) the mean fluorescence of transfected cells, and (iii) the overall mean fluorescence of the population. Highest efficiency was typically observed around day 2–3, which approximately corresponds to the time frame in which proliferation rate was the fastest according to Figure 2. Data represent mean ± SD. (B) Representative epi-fluorescent images of transfected cells on selective microcarrier. (i) FACTIII, (ii) collagen, (iii) plastic^+^, (iv) plastic, (v) glass, (vi) CultiSphere S, (vii) Pronectin F, (viii) Cytodex 3, and (ix) Hillex II. Cells were transfected 2 or 3 days after seeding. Scale bar, 500 μm.

We next transfected microcarrier culture on days 2, 3, 5, and 7 after seeding to determine whether the high-proliferative phases of the culture period corresponded to higher transfection efficiencies. Figure 3A shows the transfection efficiencies as a function of the percent of cells transfected (Figure 3A-i), the mean fluorescence of transfected cells (Figure 3A-ii), and the overall mean fluorescence of the population (Figure 3A-iii). Transfection on day 2 or 3 after seeding generally resulted in higher efficiencies than on days 5 and 7, when growth began to plateau. Microcarriers that supported the fastest proliferation rate (Cytodex 3, CultiSphere S, Hillex II, and glass) also resulted in the highest transfection efficiencies. Neither surface material nor cationic charge influenced transfection, as comparable efficiencies were observed between plastic and plastic^+^. The differences in transfection efficiencies can further be seen under epi-fluorescence microscope, as shown in Figure 3B. Cytodex3 (Figure 3B-viii) had more GFP^+^ cells across the field of view than either FACTIII (Figure 3B-i) or plastic^+^ (Figure 3B-iii), the latter of which show GFP^+^ cells that are more sparsely distributed and punctuated in fluorescent intensity. In general, microcarriers that can support higher proliferation resulted in higher overall transfection efficiencies.

#### Optimal Concentrations of Transfection Complexes

**Figure 4:**
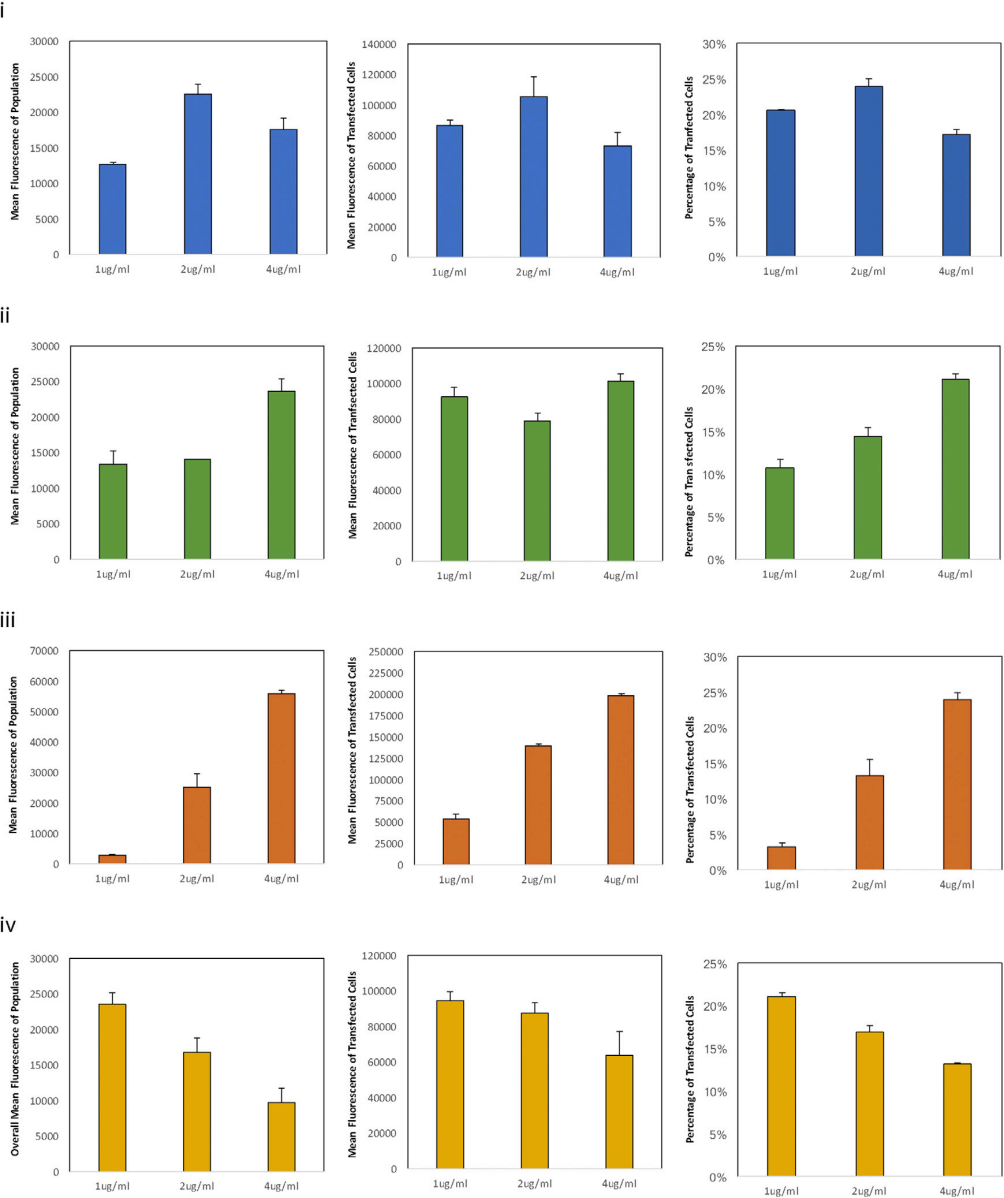
Effect of DNA Concentration on Transfection Efficiencies Cells were cultured on (i) Cytodex 3, (ii) glass, (iii) Hillex II, and (iv) CultiSphere S then transfected on day 3 after seeding. Transfection efficiencies are shown here as a function of percent of cell transfected (left column), mean fluorescence of transfected cells (middle column), and the mean fluorescence of the cell population (right column). Data represent mean ± SD.

To determine the optimal amount of DNA needed on a per-cell basis, we transfected Cytodex 3 (Figure 4-i), glass (Figure 4-ii), Hillex II (Figure 4-iii), and Cultisphere S (Figure 4-iv) at 1 μg/mL, 2 μg/mL, and 4 μg/mL. The optimal concentrations differ among microcarriers, with cells on Cytodex 3 being optimally transfected at 2 μg/mL while cells cultured on glass, CultiSphere, and Hillex II needed 4 μg /mL to reach the optimal transfection efficiencies. However, this is more reflective of the differences in cell number at the time of transfection rather than the characteristic of the microcarrier per se. That is, Cytodex had about half the number of cells at the time of transfection compared to CultiSphere, glass, or Hillex II (∼1.37 × 10^5^ versus ∼2.7 × 10^5^). This highlights an important difference in the way that cell culture components are calculated between planar culture and microcarrier suspension culture. Based on the cell number at the time of transfection and the concentration of DNA that lead to the highest efficiencies, we estimated that the optimal concentration is approximately 1 μg DNA per 2.2 × 10^5^ − 2.7 × 10^5^ cells. For this proof-of-concept study, we chose Cytodex 3 for subsequent work in stirred suspension owing to its relative ease of culturing, uniformity in transfection, consistency in cell proliferation, as well as our previous experience in growing other cell types with it.^21^

### Transfection in the Bioreactor

#### Effect of Seeding Density on Proliferation Rate

**Figure 5:**
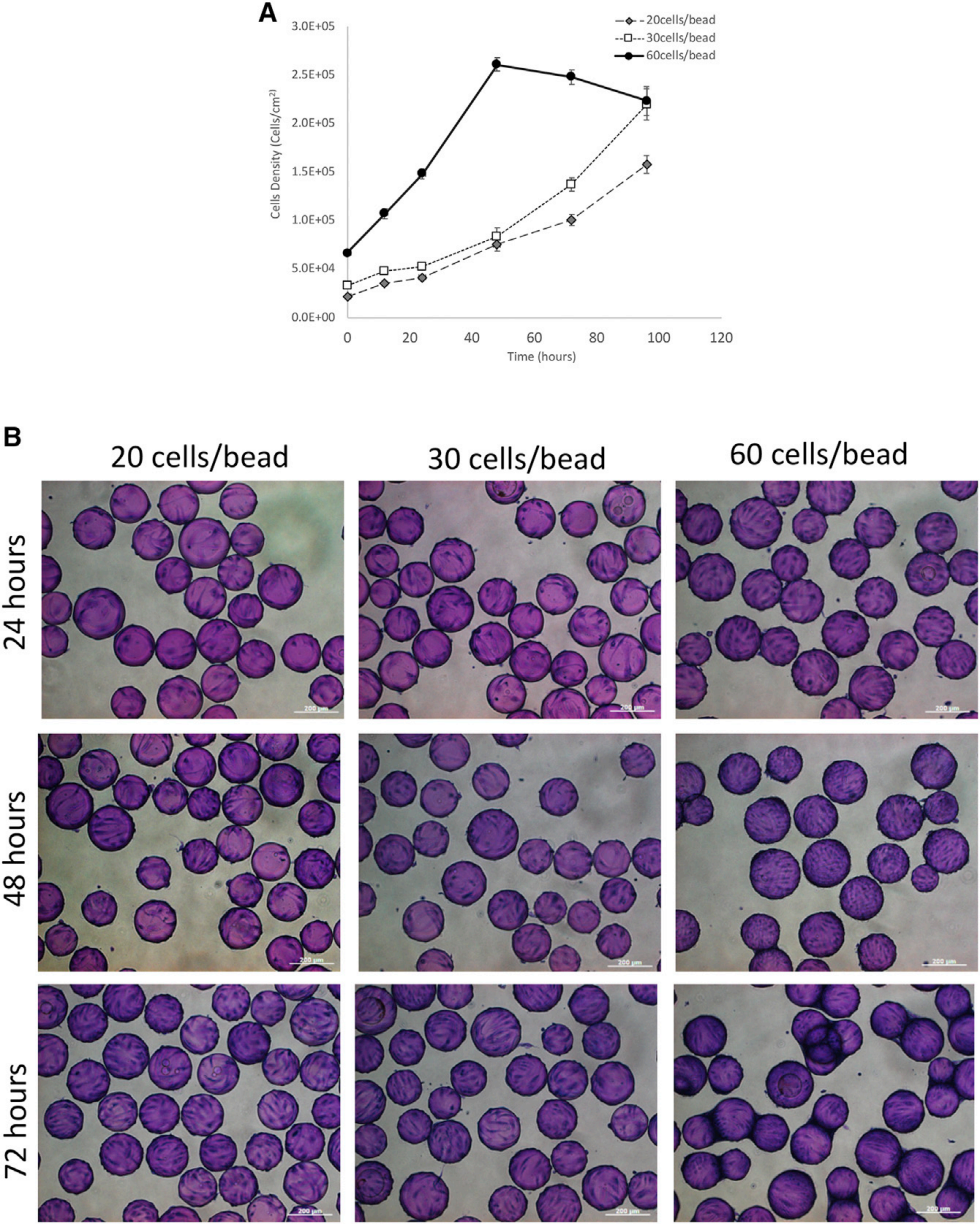
The Effects of Cell Seeding Density on the Growth of NHFF on Cytodex 3 (A) The effect of cell seeding density on the growth curve of HFF on Cytodex 3. Cells were inoculated at a density of 20 cells/bead (diamond), 30 cells/bead (open square), and 60 cells per bead (filled circle). Data represent mean ± SD. (B) Representative phase contrast micrographs of cells seeded on Cytodex 3 at various densities. Twenty cells/bead (left column), 30 cells/bead (middle column), and 60 cells/bead (right column) after 24 hr, (top row), 48 hr (middle row), and 72 hr (bottom row) of growth in stirred-suspension bioreactor. Cells were post-strained with crystal violet. Scale bar, 200 μm.

We have so far demonstrated that cells can be transfected on microcarriers in a static suspension. To adapt our small-scale multi-well format to a larger scale stirred-suspension bioreactor, we focused on inoculation densities in the 100-mL bioreactors to determine an optimal starting cell concentration that would lead to the fastest growth rate between 24 and 48 hr. Figures 5A and 5B show cell growth kinetics and the corresponding micrograph of cells seeded on Cytodex 3 microcarriers at densities of 20 cells/bead, 30 cells/bead, and 60 cells/bead under a continuous agitation speed of 60 rpm. This speed was chosen based on previous work completed with other adherent cell types using the same bioreactor vessel and culture volume.^17^, ^24^ Figure 5A shows that the growth rate for the culture seeded with 60 cells/bead was much faster than the cultures with densities of 20 or 30 cells/bead. This is evident by the slope of the growth curve with 60 cells/bead being 4× steeper than that of the latter two. However, with the higher proliferation rate, cells reached a plateau sooner at 48 hr, at which point they begin to fall off the microcarrier, as evident by the decrease in cell number thereafter. The saturation in cell density can also be seen in the micrograph shown in Figure 5B, where at the 72 hr pane, noticeable bridging and clumping were observed for cultures seeded at 60 cells/bead. Given the fastest growth between 24 and 48 hr were observed with a seeding density of 60 cells/bead, we chose this condition for subsequent transfection experiments.

#### Transfection in Stirred Suspension

**Figure 6:**
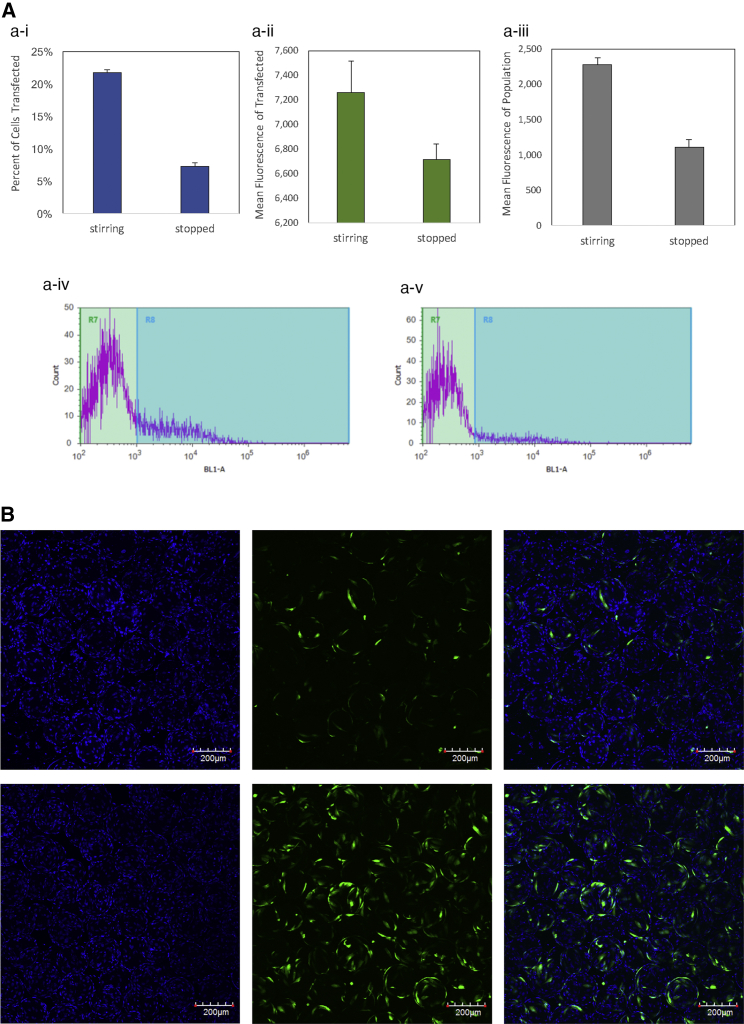
Transfection of NHFF in Suspension Bioreactor with or without Continuous Stirring (A) Transfection in the bioreactor with continuous stirring or in static suspension with stirring stopped. (i) Percentage of transfected cells, (ii) mean fluorescence of transfected cells, and (iii) overall mean fluorescence of population. Bottom panel shows fluorescence intensity histogram of cells transfected with stirring ON (iv) and with stirring stopped (v). Data represent mean ± SD. (B) Representative confocal images of cell transfection in the bioreactor without stirring (top row) or with continuous stirring (bottom row) during transfection. Left column, Hoechst stain for nucleus; middle row, GFP; right column, composite. Images are max intensity projection of approximately 325–450 stacks with 1.57 μm per stack. Scale bar, 200 μm.

To test whether mixing would enhance or interfere with transfection, complexes were added to the microcarrier suspension culture with continuous stirring and with the mixing stopped. Figure 6A shows comparison of transfection efficiencies in continuous stirred suspension (stirring) and in static suspension with agitation turned off (stopped). Not only was the percentage of cells transfected 2× higher in stirred suspension (Figure 6A-i; 21.8% versus 7.28%), but the overall mean fluorescence of the transfection cells was 2× higher as well (Figure 6A-iii; 2,271 versus 1,111). Further, the fluorescent intensities of cells transfected in stirred suspension showed a more pronounced symmetrical distribution with a center peak (Figure 6A-iv), compared to non-agitated (Figure 6A-v), which showed a flatter spread across the intensity spectrum. The differences in transfection efficiencies are further confirmed by observation under confocal microscope. Figure 6B shows max intensity projection composite images of optically sliced microcarrier culture transfected in continuous stirring (top pane) and without agitation (bottom pane). Not only were there more transfected cells visible, but the distribution of transfected cells was more even across beads. By contrast, transfection in the absence of agitation showed cells with fluorescence intensity that were unevenly distributed, with some brightly fluorescent and some dimly detectable.

#### Effects of Low-Yield Growth Conditions on Transgene Expression and Persistence

**Figure 7:**
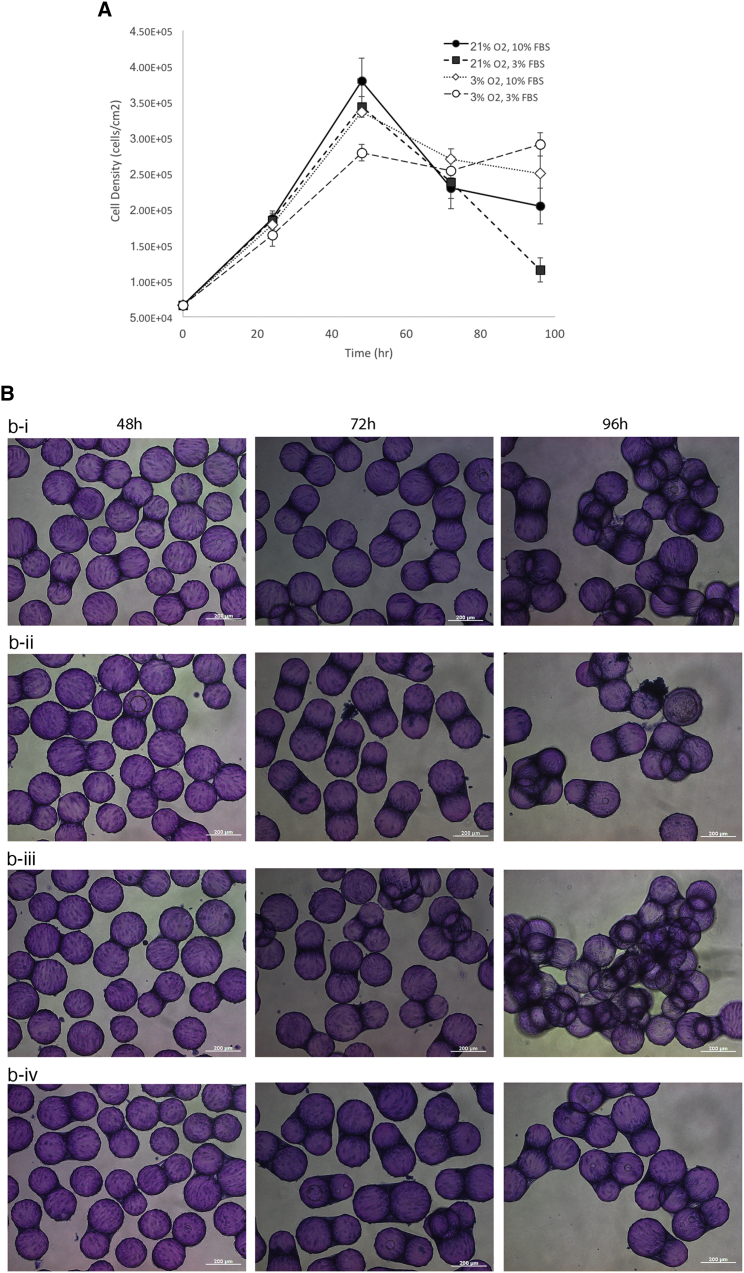
The Effect of Serum Concentration and Oxygen Saturation on the Growth of NHFF on Cytodex 3 (A) The effect of serum and dissolved oxygen concentration on the growth kinetics of HFF on Cytodex 3. Microcarrier culture were grown in stirred suspension at 21% O_2_ with 10% FBS (filled circle), 21% O_2_ with 3% FBS (filled square), 3% O_2_ with 10% FBS (open diamond), and 3% O_2_ with 3% FBS media (open circle). Data represent mean ± SD. (B) Representative phase contrast micrograph of cells grown on Cytodex 3 in stirred-suspension bioreactor. (i) Twenty-one percent O_2_ with 10% FBS media, (ii) 21% O_2_ with 3% FBS, (iii) 3% O_2_ with 10% FBS, and (iv) 3% O_2_ with 3% FBS media. Cells began to clump 48 hr into the culture. After 96 hr, significant bridging between microcarriers can be seen, at which point cells either fall off or die off. Scale bar, 200 μm.

**Figure 8:**
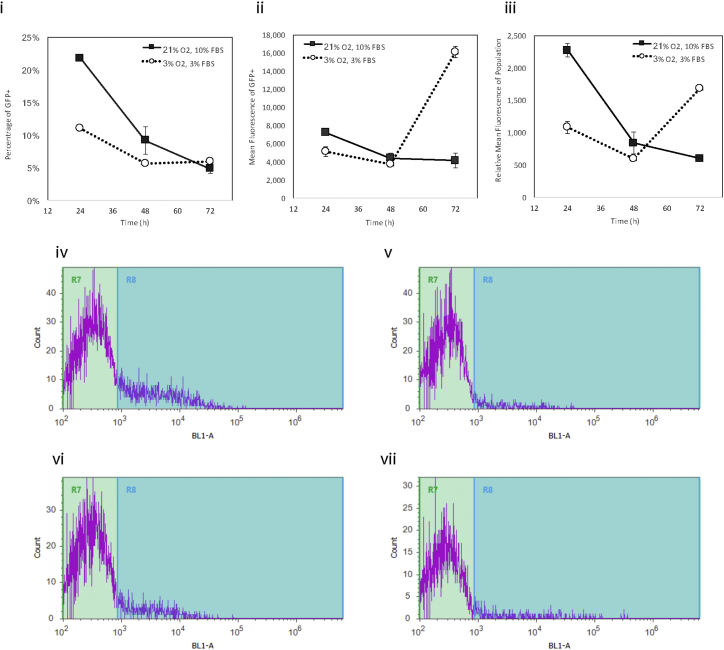
Transfection Efficiency and Level of Transgene Expression over Time under Normal Proliferation Conditions versus Low-Yield-Growth Conditions (i) Percentage of transfected cells, (ii) mean fluorescence of transfected cells, and (iii) overall mean fluorescence of population. Bottom two rows show fluorescent intensity histogram of cells transfected at 21% O_2_ and 10% FBS, on (iv) day 1, (v) day 3, and transfected at 3% O_2_ with 3% FBS at (vi) day 1 and at (vii) day 3. Data represent mean ± SD.

Non-viral transfection is a transient process with transgene expression lasting only a few days. Part of the lack of sustained transgene level is attributed to the serial dilution of the plasmid DNA and the expressed transgene through successive rounds of cell division. Thus, one way to enhance transgene persistence is to attenuate cell growth to prevent dilution of the transgene. To test this hypothesis, we cultured cells in either 21% oxygen with 10% fetal bovine serum (FBS) (normal), 21% oxygen with 3% FBS (intermediate), 3% oxygen with 10% FBS (intermediate), and 3% oxygen with 3% FBS (low yield). Figure 7 shows the growth kinetics (Figure 7A) and representative micrograph of culture grown in these conditions. Not surprisingly, cells cultured in normal conditions had the highest cell numbers after 48 hr, while cells cultured in reduced oxygen and FBS had the lowest cell count 48 hr after seeding. We then transfected cells in high-yield and low-yield conditions and found that transfected cells in the former setup quickly dropped to near-background level of fluorescence after just 72 hr (Figure 8, solid square). In contrast, cells transfected in low-yield condition had lower efficiency initially, but the level of transgene expression actually went up to 4× to that of cells transfected in normal conditions. This does not appear to be an artifact, as we see more cell count registered toward upper (toward the right) end of the fluorescent intensity histogram (Figure 8-vii). Taken together, these results suggest that transfection efficiency and transgene persistence can be enhanced and tuned by adjusting the proliferation rate.

## Discussion

Current manufacturing practices for engineered CTP is largely inherited from the process previously established for biopharmaceutics, wherein cells are manipulated in a planar tissue culture dish first, followed by bioprocess development into a scalable platform.^25^, ^26^ This “two-step” approach can be costly, labor intensive, and time consuming, particularly for cell therapy products that need to be individually sourced from patients or compatible donors. In that sense, biomanufacturing of cell therapy products requires a mass customization platform that could scale out production of custom made-to-order products individualized to the patients and their treatment requirement. This would involve combining some of the intervening manual steps into one fully integrated, enclosed, scalable, and continuous system. However, one of the biggest technical hurdles remains to be the ability to transfect cells efficiently in the bioreactor. While direct transfection of primary cells in suspension culture has been demonstrated previously, the efficiencies were comparatively lower than their planar counterpart.^27^ Interestingly, what we found in this study is that transfection in the stirred-suspension environment is not only feasible, but more efficient than planar culture, since cells grew faster and more uniform in the bioreactor. The efficiency we achieved with non-viral vectors is comparable to previous reports of viral transduction of T cells in suspension culture^4^, ^27^ and more than 10× higher than liposomal reagent reported elsewhere.^28^ We were able to achieve efficient transfection using only off-the-shelf, commercially available reagents by empirically determining the most conducive transfection pairing of reagent/microcarrier, then titrating the culture conditions to maximize the utility of the transfection complexes, all without the use of any chemical modification, genetic enhancer, novel conjugates, inhibitors, or additives. Incidentally, the high proliferative rate conducive to transfection may have inadvertently accelerated the decline in transgene persistence, since subsequent rounds of cell division following the initial transfection event effectively diluted transgene among daughter cells. As well, non-transfected cells likely outgrow the transfected cells at a faster rate, proportionally decreasing the overall percentage of transfected cells. In that sense, we anticipate overall transfection efficiency could be even higher and more sustained if the proliferation rate could be more precisely modulated in a fully computerized system where culture conditions can be monitored and dynamically controlled.

The advantage of engineering cells directly in the bioreactor extends beyond the potential for a fully integrated, scalable, and controlled system. It is well known that mechanical and physical cues can regulate cell fate and cell behavior during developmental processes in both stem and adult cells.^29^ In that regard, the hydrodynamic forces within the stirred-suspension environment can be further harnessed to modulate cellular processes to enhance the derivation and expansion of cell therapy products.^30^, ^31^ We have previously shown that induction of pluripotency in the stirred-suspension bioreactor is two orders of magnitude higher than in planar static culture,^32^ which we postulate were partly attributed to cellular responses to the fluid shear stress that concomitantly resulted in the activation of pluripotency genes via a common mechanotransduction pathway.^33^ Regardless of what the underlying processes were that lead to the increase in reprogramming efficiency, it is clear that cellular responses to the hydrodynamic environment in the bioreactor can either enhance or antagonize the engineered phenotype. As such, it is critical that bioprocess be incorporated into the development of CTP early on, such that the response to microenvironment can be fully integrated into the overall system design.

Transfection and transduction efficiencies are often viewed as a key rate-limiting step from a manufacturing point of view,^4^ which has so far limited its integration into the bioprocess development. We hope our transfection method here will encourage researchers to further explore the utility of direct transfection in suspension bioreactors and adopt the bioreactor platform into their somatic cell reprogramming strategies to streamline the translation of engineered CTP into clinical practice.

## Materials and Methods

### Cell Culture

Previously derived HFFs^34^ were cultured in DMEM (Lonza) with high D-glucose and L-glutamine, supplemented with 10% heat-inactivated FBS (Gibco), 0.1 mM DMEM non-essential amino acids, 50 U/mL penicillin, and 50 mg/L of streptomycin. Cells were maintained in a humidified 37°C incubator with 5% CO_2_. Cells between passage 4 and 12 generations were used in this study.

### HFF Microcarrier Culture

PolyGEM polystyrene microcarriers (Hillex II, FACTIII, plastic, plastic^+^, and collagen) were obtained from Global Cell Solutions. Cytodex 3 and CultiSphere S were from Sigma. Each of the microcarriers were prepared for cell culture according to the manufacturer’s recommendation. In brief, microcarriers were weighed and hydrated in 50 mL of either Ca^2+^ and Mg^2+^ free PBS (CMF-dPBS; Lonza) or deionized water overnight at room temperature, according to manufacturer’s suggestion. After 24 hr, the supernatant was removed and the microcarriers were washed twice in fresh PBS then sterilized by autoclaving at 120°C/23.25 psi for 25 min. After sterilization, microcarriers were equilibrated in 50 mL of DMEM.

To prepare microcarrier culture in static suspension, multi-well tissue culture treated plates were first coated with the hydrogel Polyhydroxyethylmethacrylate (pHEMA) to prevent fibroblasts from attaching to the plate. pHEMA is prepared by dissolving the hydrogel crystal in 100% ethanol (EtOH) at a final concentration of 20 mg/mL overnight at 42°C with agitation. Plate coating was done by rinsing the tissue culture surface with pHEMA and allowing it to equilibrate for 5 min inside the incubator at 37°C; excess pHEMA was then drawn out and discarded. Freshly coated plates were then left in the incubator overnight to allow excess ethanol to evaporate. Once dried, microcarriers were dispensed into each well such that the microcarriers covered the entire surface area, then incubated at 37°C for at least 2 hr to equilibrate the microcarriers for optimal attachment.

To seed cells on microcarriers, a confluent static culture of HFF were washed 2× with CMF-dPBS; cells were then detached by TrypLE Express (Gibco) and triturated into single-cell suspension. Approximately 50,000 cells were then dispensed into each well of a 48-well plate covered with microcarriers. Inoculated microcarrier cultures were returned to the incubator; plates were intermittently agitated every 10–15 min to disperse the microcarriers and the cells for up to 1 hr to maximize attachment efficiency and uniformity.

### Growth Curve on Microcarrier by MTT Assay

Proliferation of viable cells were indirectly measured using the 3-(4,5-dimethylthiazol-2-yl)-2,5-diphenyltetrazolium bromide (MTT) assay (Molecular Probes), wherein the yellow tetrazolium salt was reduced in metabolically active cells to form insoluble purple formazan crystals. To process cells for viability measurement at each time point, a solution of MTT was added directly to the medium to a final concentration of 1 mg/mL and incubated at 37°C for 2 hr; supernatant was then removed by aspiration. Cells with internalized MTT crystals on the microcarrier were then solubilized by the addition of acidic isopropanol (isopropanol + 10% Triton X-100 and 1 drop of concentrated hydrochloric acid [HCL] per 50 mL of solution). The absorbance was measured at 570 nm with the background subtracted at 650 nm using the Bio-Rad Benchmark Plus UV/Vis Microplate Reader. Absorbance readings were then expressed as a percentage of static culture that were mitotically arrested cells by treatment with 10 μg/mL of mitomycin C (Sigma) for 4 hr at the time of seeding to normalize the day-to-day variation in absorbance.

### Transfection of HFF

The gWIZ-GFP (Alvedron) plasmid is a 5,757-bp mammalian expression plasmid that contains a modified promoter from the human cytomegalovirus immediate-early genes. pCE-GFP were obtained from Addgene (access no. 41858). Plasmid DNA was transformed into *Escherichia coli* (DH5α; Life Technologies, Ontario, Canada) then grown in Luria-Broth supplemented with 30 μg/mL kanamycin as previous described;^35^ transfection-grade plasmid DNAs were then purified from the transformed bacteria using the PureLink HiPure Plasmid Midiprep Kit, according to manufacturer’s protocol, with the modifications that all reagents were pre-chilled on ice prior to performing all subsequent procedures at 4°C.

Cells were transfected with either XtremeGENE HP DNA Transfection Reagent (Sigma), TransIT-LT1, TransIT-2020, TransIT-X2, TransIT-3D (Mirus Bio), or JetPrime (Polypus) according to manufacturer’s recommended protocol. In brief, plasmid DNA was diluted in OPTI-MEM (Gibco) at a concentration of 10 μg/mL, then transfection reagent was added at a specified volume-to-weight ratio (v/w) in drop-wise fashion, vortexed immediately, incubated at room temperature for 16 min, then diluted in basic media with 10% FBS to a final plasmid DNA concentration of 1 μg/mL. Cells were then incubated overnight, up to 24 hr, at which point reporter gene expression could be observed or quantitated by either epi-fluorescent microscope or by flow cytometry.

### Analysis of Transfection Efficiency by Flow Cytometry

Transfection efficiency was assayed by fluorescence-activated cell sorting (FACS) as previously described.^36^ In brief, cells were washed 3× with CMF-dPBS for 5 min, then detached from culture substrate using with 1× TrypLE Express (Gibco, Gaithersburg, MD, USA) and subsequently dissociated into single-cell suspension to be fixed in 3.7% formaldehyde in CMF-dPBS.

To process transfected cells on microcarriers for flow cytometry, an aliquot of the microcarrier culture was drawn out from the bioreactor and transferred to a clean tube. Cells were dissociated from the microcarrier as per above for static culture, except 0.25% Trypsin-EDTA was used instead; dissociated single-cell suspension were subsequently passed through a 40 μm cell strain prior to analysis. Samples were subjected to FACS using an Attune Acoustic Focusing Cytometer (Thermo Fischer Scientific) equipped with a 488 nm and 637 nm laser and analyzed on the Attune Software (v2.1.0). A minimum of 5,000 events were collected per sample. Analysis of intact viable cells was performed by gating the appropriate area and width of side and forward scatter to avoid cellular debris; transfection efficiency analysis was then performed by gating the fluorescent intensity of the cell population in the BL1 channel (excitation [ex] 488 nm/emission [em] 525 nm) such that the negative control (i.e., cells transfected with blank expression plasmid gWIZ) had 1%–2% autofluorescent cells.

### Microcarrier Preparation for Bioreactor Culture

Methods for culturing cells on microcarriers in stirred-suspension bioreactors was carried out as described previously.^37^ In brief, Cytodex 3 microcarriers (GE Healthcare Life Sciences) were used for all bioreactor experiments. Before the microcarriers were seeded into the 100 mL stirred-suspension bioreactors (Corning), they were hydrated, washed, and autoclaved. The desired amount of microcarriers were weighed and added to a siliconized 125 mL Erlenmeyer flask with 100 mL of Ca^+^/Mg^+^ free PBS (Life Technologies) containing 1% Antibiotic-Antimycotic (Anti-Anti, Life Technologies). Each bioreactor was inoculated with 2 g/L of microcarriers. Three drops of Tween 80 (United States Chemical Corporation) was added into the flask to lower the surface tension and prevent the microcarriers from sitting at the top of the liquid. The microcarriers were left to hydrate at room temperature for a minimum of 6 hr. After hydrating, 80 mL of the PBS solution was aspirated out with a 25-mL pipette, leaving 20 mL of the PBS solution in the flask with the microcarriers. Next, 25 mL of fresh PBS with 1% Anti-Anti was added to the flask. The microcarriers were settled for 5 min, then 25 mL of the PBS solution was aspirated out and discarded. This washing procedure was repeated three times. During the final washing step, 30 mL of PBS was added to the Erlenmeyer flask, resulting in a total volume of 50 mL. The Erlenmeyer flask was then sealed with parafilm and placed in a 4°C fridge overnight. Before inoculation, the microcarriers were autoclaved using a liquid cycle. The PBS solution was then removed and DMEM was added to the microcarriers using a 10-mL pipette. For each bioreactor being inoculated, 20 mL of DMEM was added. The microcarriers were then split into 50-mL conical tubes (FroggaBio). Each conical tube was used to inoculate one bioreactor. The microcarriers were settled in the conical tubes, and the DMEM was aspirated out. Finally, the microcarriers were seeded into siliconized bioreactors with 60 mL of medium each. The bioreactors were placed in the incubator on a magnetic stir plate set at 60 rpm to acclimatize overnight.

### Inoculating Microcarrier for Stirred-Suspension Bioreactor

HFFs were inoculated into the bioreactors from static 100-mL culture dishes (VWR) on day 4 of culture. To passage the static culture cells, the medium was first aspirated from the culture dish. The cells were then washed twice with 5 mL of Ca^+^/Mg^+^ free PBS with 1% Anti-Anti. To cleave the cells, 3 mL of TrypLE (Thermo Fisher Scientific) was added, and the culture dish was placed in the incubator at 37°C for 5 min. The cells and TrypLE were then removed using a 5-mL pipette and added to a 15-mL conical tube (FroggaBio). The culture dish was washed with 5 mL of medium, which was added to the conical tube to be centrifuged at 300 × *g* for 5 min. The supernatant was then aspirated off, and the cell pellet was broken apart by adding 1 mL of medium and triturating 5 times with a 1,000-μL pipette. Additional medium was added to the conical tube, and samples were taken for counts using the NucleoCounter (ChemMetec) automatic cell counter. An average cell density was used to seed the bioreactors at desired cell concentrations. Culture medium was then added to the bioreactors so that each vessel had a total volume of 100 mL. The bioreactors were then placed back on the magnetic stir plates in the incubator.

### Cell Growth Kinetics in Stirred-Suspension Bioreactors

Various cell concentrations were tested to determine an optimum inoculation density that would result in the fastest growth rate within 48 hr of seeding. Bioreactors were inoculated at concentrations of 20, 30, and 60 cells/bead. Cell counts and images were taken at 12 hr, 24 hr, then every 24 hr following for a total of 4 days. For each condition, two bioreactors were inoculated, and two 3-mL samples were taken from each bioreactor at each time point to count. Each 3-mL sample was put into a 15-mL conical tube, and the microcarriers were settled for 5 min. The supernatant was removed and the microcarriers were washed three times with 1 mL of Ca^+^/Mg^+^ free PBS with 1% Anti-Anti solution as described previously. The cells were then re-suspended in 1 mL of PBS, and two 100-μL samples were taken from each conical tube to be counted with the NucleoCounter. Images of the cells on the microcarriers were taken with a Zeiss Axiovert 25 microscope (Carl Zeiss). A 0.5-mL sample was removed from each bioreactor using a 5-mL pipette and added to a 6-well plate with 1.5 mL of PBS. Twenty microliters of 0.5% crystal violet (Sigma Aldrich) in methanol was added to each well and left to sit for 5 min at room temperature. Images were taken at 10× magnification with the filter set to phase 0, as this gives a flatter image that refracts less light making the cells easier to see.^8^

### Optimizing Growth Conditions for Transfection in Stirred-Suspension Bioreactors

To slow down cell division for post-transfection efficiency and reduce microcarrier clumping at high cell densities, different environmental conditions were tested using a factorial design experiment. The atmospheric O_2_ concentration and percent of FBS in the medium were altered, and cell counts and images were performed every 24 hr for a total of 4 days. Bioreactors were seeded at 60 cells/bead and placed in the incubator for 24 hr. At 24 hr, two bioreactors were changed to each of the new conditions: 21% O_2_ and 3% FBS, 3% O_2_ and 10% FBS, and 3% O_2_ and 3% FBS, to be compared with the original condition of 21% O_2_ and 10% FBS.

### Immunofluorescence and Confocal Microscopy

Aliquots of HFF microcarrier culture were collected and fixed in 3.7% formalin in CMF-PBS for 1 hr at room temperature. Microcarriers were dispensed into a glass-covered dish and imaged using an Olympus IX81 FV1000 Laser Scanning Confocal equipped with 405 nm, 488 nm, 559 nm, and 635 nm lasers and corresponding emission filter sets. Post-acquisition image analysis was done using FluoView (Carl Zeiss AG, Oberkochen, Germany) and ImageJ.

### Statistical Analysis

Where indicated, the data is summarized as the mean ± SD of triplicate measurements. Unpaired Student’s t tests were used to assess statistical differences (p < 0.05) between the group means. All experiments were done in triplicate with a minimum of three independent experiments.

## Author Contributions

Conceptualization, C.Y.M.H.; Methodology, C.Y.M.H., T.W., B.S.B.; Investigation, C.Y.M.H., T.W., B.S.B.; Writing – Original Draft, C.Y.M.H.; Writing – Review & Editing, C.Y.M.H., T.W., B.S.B., M.S.K., D.E.R.; Funding Acquisition, M.S.K., D.E.R.; Supervision, M.S.K., D.E.R.
